# VLP-Based Vaccines as a Suitable Technology to Target Trypanosomatid Diseases

**DOI:** 10.3390/vaccines9030220

**Published:** 2021-03-05

**Authors:** Aline Maria Vasconcelos Queiroz, Johny Wysllas de Freitas Oliveira, Cláudia Jassica Moreno, Diego M. A. Guérin, Marcelo Sousa Silva

**Affiliations:** 1 Immunoparasitology Laboratory, Department of Clinical and Toxicological Analysis, Federal University of Rio Grande do Norte, Natal 59078-970, Brazil; alinemariavq@gmail.com (A.M.V.Q.); johny3355@hotmail.com (J.W.d.F.O.); claudia.mrn1@gmail.com (C.J.M.); 2 Postgraduate Program in Pharmaceutical Sciences, Federal University of Rio Grande do Norte, Natal 59078-970, Brazil; 3 Postgraduate Program in Biochemistry, Federal University of Rio Grande do Norte, Natal 59078-970, Brazil; 4 Instituto Biofisika (CSIC-UPV/EHU), Department of Biochemistry and Molecular Biology, University of the Basque Country, 48940 Bizkaia, Spain; 5 Global Health and Tropical Medicine, Institute of Hygiene and Tropical Medicine, New University of Lisbon, 1349-008 Lisbon, Portugal

**Keywords:** virus-like particles, vaccine, trypanosomatids, Chagas disease, leishmaniasis, African trypanosomiasis

## Abstract

Research on vaccines against trypanosomatids, a family of protozoa that cause neglected tropical diseases, such as Chagas disease, leishmaniasis, and sleeping sickness, is a current need. Today, according to modern vaccinology, virus-like particle (VLP) technology is involved in many vaccines, including those undergoing studies related to COVID-19. The potential use of VLPs as vaccine adjuvants opens an opportunity for the use of protozoan antigens for the development of vaccines against diseases caused by *Trypanosoma cruzi*, *Leishmania* spp., and *Trypanosoma brucei*. In this context, it is important to consider the evasion mechanisms of these protozoa in the host and the antigens involved in the mechanisms of the parasite–host interaction. Thus, the immunostimulatory properties of VLPs can be part of an important strategy for the development and evaluation of new vaccines. This work aims to highlight the potential of VLPs as vaccine adjuvants for the development of immunity in complex diseases, specifically in the context of tropical diseases caused by trypanosomatids.

## 1. Introduction

**Figure 1:**
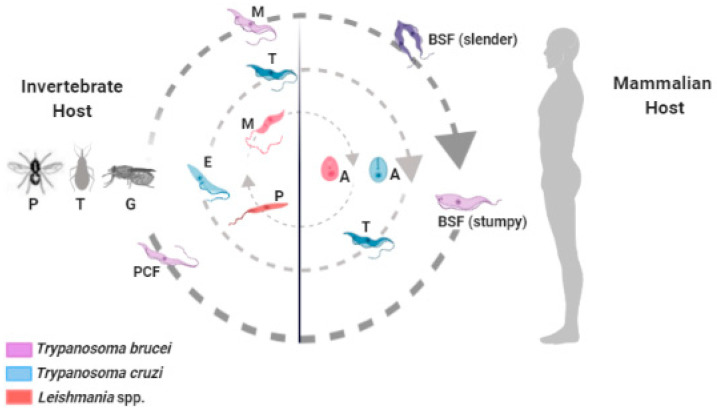
Schematic representation of the biological cycle of some trypanosomatids: *Trypanosoma brucei* (lilac), *Trypanosoma cruzi* (blue), and *Leishmania* spp. (red). Invertebrate hosts: *Phebotomineos* (P), *Triatoma* sp. (T), and *Glossina* sp. (G). The different evolutionary forms of these protozoa are represented: bloodstream form (BSF); procyclic form (PCF); metacyclic forms (M); amastigotes (A); promastigotes (P); epimastigotes (E); and trypomastigotes (T).

*Trypanosoma cruzi*, *Trypanosoma brucei*, and *Leishmania* spp. are flagellate parasites that belong to the Trypanosomatidae family and have a complex life cycle between invertebrate and mammalian hosts, where they cause Chagas disease, African trypanosomiasis, and leishmaniasis, respectively (Figure 1). These diseases mainly affect economically vulnerable populations, leading to thousands of annual deaths and major public health problems [1].

To ensure survival during infection, these protozoa have developed sophisticated evasion mechanisms to circumvent the immune system and virulence factors that facilitate the maintenance of the parasite–host interaction. Their invasion and embedment in various tissues lead to the establishment of the chronicity of these diseases. By invading specific tissues and regions of the body and staying within them, parasites hamper the accessibility of drugs that are traditionally used to treat these diseases, which leads to low efficiency in the chronic stage and, in many cases, high toxicity. Therefore, the mammalian inability to eliminate these parasites efficiently and the lack of adequate treatment require the search for new technologies to prevent and combat these infectious diseases [2,3,4].

In this scenario, the development of vaccines to control these infectious diseases, despite representing a major challenge from a biotechnological point of view, is an urgent need. Due to the biological complexity of these protozoa, characterized mainly by different evolutionary forms, the search for antigenic candidates for the design of vaccine prototypes represents a major issue. On the other hand, during the maintenance of the infection, these parasites cause immunity response polarization, a phenomenon that governs the resistance and susceptibility mechanisms of the infected hosts. Thus, the application of technology involving virus-like particles (VLPs) has become an interesting tool for the development of vaccines in the context of infections caused by trypanosomatids, since VLPs have the immunoadjuvant ability to manipulate immune response polarization [5].

VLPs used as vaccine adjuvants provide an alternative to current chemical adjuvants, along with an ability to carry foreign epitopes; they constitute platforms for the development of new prototypes and vaccine protocols. VLPs’ immunostimulatory properties are similar to natural infections of the viruses from which they are derived, and the preservation of the native conformation is, in addition to the primary sequence, a major factor responsible for this property [6,7]. Therefore, parasitic antigens presented to the immune system in the context of VLPs (chemical linkage or genetic manipulation) represent an interesting biomedical application for the development of state-of-the-art vaccines. Thus, the objective of this work is to reflect on the use of VLPs as vaccine adjuvants in the context of infections by trypanosomatids of medical importance.

## 2. Application of the Use of VLPs in the Development of New Vaccines

The structural conformation of VLPs mimics the morphology and structures of viral particles without the presence of the virus genome, and therefore, they are not infectious particles. Thus, the main advantages of using VLPs as vaccine prototypes are safety, since they are not infectious viral particles, and the ability to induce a robust immune response that is highly similar to that elicited by a natural viral infection. These two characteristics allow VLPs to be used in the development of vaccines [8].

Additionally, there are several ways to produce recombinant VLPs, mainly using gene expression systems in bacteria, yeasts, insect cells, and mammalian cells, among others [9]. The strategy for the construction and production of recombinant VLPs depends on viral biology, mainly the presence or absence of a viral envelope, and the choice of the best system for gene expression and purification of recombinant particles [10].

Regarding VLP-based vaccines, about a dozen are currently approved and available in Europe and the USA. These include Recombivax HB^®^ [11] and Engerix^®^, both against hepatitis B [12], and Gardasil^®^ against human papillomavirus (HPV) [13]. Cervarix™ against HPV is relevant to highlight, which contains VLPs produced in insect cells used in the vaccine market [14,15]. Regulatory agencies have recognized and approved this production system for its capacity to generate biotechnological products in a safe, effective, and economical way [16]. 

During the past few decades, studies on different VLP systems used against important human diseases—such as Ebola [17], Chikungunya [18], Dengue [19], influenza A [20], respiratory syncytial virus [21], and Zika [22]—as well as a therapeutic strategy for cancer of the breast [23], have demonstrated the potential of this technology for the production of new vaccines. More recently, two of the 64 vaccine projects in progress are based on VLPs, including those related to the COVID-19 pandemic [24]. This demonstrates the potential and popularization of the use of VLPs for the development of new vaccines.

In the context of the use of VLPs for the development of vaccines against protozoa, it was observed that the immunostimulatory property of VLPs with targeted epitopes is able to induce the production of protective antibodies and a high survival rate of animals infected with *Toxoplasma gondii*, the etiologic agent of toxoplasmosis [25,26]. In the context of malaria, caused by a protozoan of the genus *Plasmodium*, the presence of lasting immunity is observed when different vaccine prototypes are produced using antigens associated with VLPs [27,28,29].

## 3. Vaccines to Control Diseases Caused by Trypanosomatids: What Do We Need and Where Are We?

The main challenge in the development of trypanosomatid vaccines is the adequate identification of important antigenic candidates for the generation and maintenance of protective immunity. A second challenge is related to the type and nature of the immune response induced during the immunization process. However, several studies have been performed in order to identify potential antigenic candidates for these protozoa, as well as mechanisms of activation of immunity for the development of vaccines (reviewed in Reference [30]). Therefore, understanding the cellular and molecular biology of these protozoa becomes essential to guide new studies in the field of vaccinology to control these parasitic diseases.

Currently, several tools are being used in the development of vaccines in the context of trypanosomatids, such as immunoinformatic, genomic, and proteomic techniques that mainly contribute to the identification of new antigenic targets and epitopes of these parasites [31,32], as well as in the development of various immunization protocols in experimental models [33,34,35,36,37,38]. Several proteins and virulence factors are biologically and structurally shared among protozoa of the trypanosomatid family and therefore could be used as possible targets for the development of vaccines, such as surface glycoproteins [39,40,41], cysteine proteases [42,43], and metalloproteases [44,45,46].

Additionally, for the development of vaccines against these trypanosomatids of medical importance, VLPs are presented as alternatives to induce immunity in a polarized way, since the mechanisms of resistance and susceptibilities of these infections are related to the immune response polarization that occurs during a natural infection. Thus, due to their constitution, they may have the ability to express specific components in amounts comparable to high quantities of these parasites and, furthermore, can present other immunogenic components, thus allowing a stronger general immune response of the organism [47].

The immune response polarization of the Th1 type (CD4^+^ T helper cells) is important for controlling infections caused by trypanosomatids, mainly due to the production of interferon-γ (IFN-γ) and tumor necrosis factor α (TNF-α). These cytokines induce the activation of macrophages and, consequently, an increase in the production of reactive oxygen species, which control the parasitic load during infection. Trypanosomatid parasites have the ability to reduce Th1-type immune response polarization by enhancing the production of IL-10 and IL-17 cytokines due to the release of numerous virulence factors [48]. On the other hand, the polarization of the Th2-type immune response is responsible for triggering the polyclonal activation of B cells that result in hypergammaglobulinemia, a phenomenon characterized by the increased production of antibodies with low specificity and affinity [49]. Therefore, during the parasite–host interaction, trypanosomatids have the ability to cause immune response polarization, which facilitates the parasitism relationship.

Despite the importance of VLP-based vaccines as a strategy to induce Th1-type immune response polarization, this methodology is still poorly studied in trypanosomatids. Indeed, only one study was found on Chagas disease [50], and only two used leishmaniasis [51,52] models. However, to date, no study has been presented in the context of African trypanosomiasis.

In recent decades, several studies have demonstrated the ability of VLPs to stimulate the activation of B cells and dendritic cells in the context of MHC class II and, consequently, the production of cytokines related to the activation of CD4^+^ T helper cells, which strongly favors the induction of humoral and cellular immunity [8,53]. In contrast, the presentation of antigens in the context of MHC class I favors the induction of cellular immunity mediated by CD8^+^ T cells and favors the induction of Th1 cells [54]. This phenomenon of immune response polarization (Th1 versus Th2) is an important opportunity for biomedical research, since the mechanisms of resistance and susceptibility to infections caused by trypanosomatids depend on the immune response polarization. The importance of the Th1 immunity profile during infections by *T. cruzi* and *Leishmania* spp. is due to the intracellular forms of these parasites during parasitism in the host [55]. In contrast, *T. brucei* does not develop an intracellular phase of infection; however, this protozoan has an antigenic variation mechanism that favors the chronicity of the infection in the host.

## 4. Development of Vaccines Based on VLPs: A Checklist

**Figure 2:**
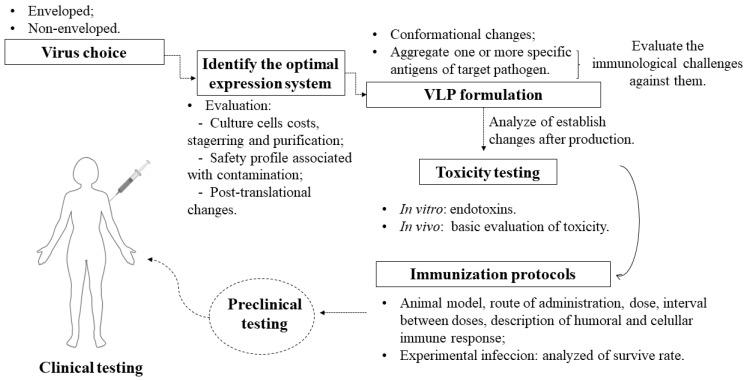
Representation of a rational scheme for the development of new vaccines based on virus-like particles (VLPs).

Based on the previous discussion, for the use of VLPs as a strategy for the development of vaccines against trypanosomatids, some criteria must be considered, represented schematically in Figure 2.

According to the illustration, there are some essential steps in the checklist:

1. Should the chosen virus be enveloped or not? Enveloped VLPs are inherited from structured viral proteins, thus generating a particle with target antigens integrated on the external surface, whereas non-enveloped VLPs are based on the expression of one or more necessary viral proteins without inheriting them from a host [56]. Both have the additional possibility to integrate antigens [57] and additional adjuvants [7] in vaccine formulations.
2. Which expression system is ideal? It should be noted that, depending on the system, there will be limitations such as cost and difficulty in scheduling [58], as well as a higher level of purification when faced with contamination from cells [59]. Some systems differ from mammalian cells mainly in the protein glycosylation phase [60] and do not give rise to a eukaryotic expression environment. The insect cell system has come to solve some problems in the production of particulates, as it has characteristics such as a eukaryotic environment, which is required for the glycosylation of proteins: this post-translational characteristic is also found in human cells, but the cost of their application is higher [61]. In addition, there is malleability in large-scale cultivation and the possibility of using it for the simultaneous expression of many proteins that facilitate the assembly of VLPs, which requires a structure with a high level of safety and a special culture system [58].
3. The structural modification of the conformational and immunological characteristics of the VLP can adjust the relative degree of the immune response for a balanced induction of humoral and cellular immunity [62], thereby generating an improved candidate capable of redirecting the immune response against specific targets when combined with antigens [63]. Thus, this modulates the balance of Th1 and Th2 immune responses to increase the specificity and affinity towards the parasite [48]. Therefore, conformational changes and the aggregation of antigens are necessary to analyze changes in stability that can compromise the effectiveness of VLP vaccines [64].
4. Before choosing the antigens, it is necessary to assess the immunological challenges found in the parasites that cause Chagas disease, leishmaniasis, and sleeping sickness. They have specific mechanisms of escape from the immune system that end up reducing the specific response of the immune system. Based on this assessment, one can then envision a single vaccine for three diseases or specific vaccines for each one, as VLPs will act as transporters of antigens, with greater security than using soluble antigens [6]. The applicability of antigens presented in various evolutionary forms of the protozoan, as well as the attempt to administer immunizations with more than one antigen to cover all forms of life and increase the antigenic repertoire, must be considered in studies on *T. cruzi*, given the genetic variability of this parasite [65]. In addition, the use of the same antigens for both prophylactic and therapeutic strategies should be considered, taking into account that prophylactic and therapeutic vaccines can prevent infection and interfere with the progression of infection, respectively. For an infected person, a therapeutic vaccine with or without an association with available drugs can have a significant impact on preventing complications, as has already been reported for Chagas disease [66].
5. After the production and necessary testing of these particles in isolation and in association with antigens, in vitro and in vivo tests need to be performed, for example, endotoxin evaluation [67]. In addition, a basic toxicity assessment must be performed, according to the WHO guidelines for non-clinical evaluation of vaccines [68]. This step can also assist in understanding the humoral and cellular immunogenicity of the isolated particles without antigen association. The choice of adjuvant must include consideration of the cost–benefit trade-off of co-stimulation, as VLPs can assume the role of self-adjuvant due to their particulate and multivalent nature, causing efficient incorporation by antigen-presenting cells (APCs) [69,70].
6. In vivo tests on immunization strategies with these specific antigens and/or with a chimeric VLP should be performed by firstly establishing the animal model, time, and doses, characterizing the humoral and cellular immune responses, and importantly, considering different routes of administration to understand whether this influences the stability of VLPs [16].
7. Viral challenges in animals (experimental infection) used in experiments are necessary to understand, for example, whether the formulation of specific antigens with VLP technology helps or not in the survival of those infected. After pre-clinical trials proceed as expected, clinical trials have a significant step in producing results that confirm a specific, lasting, and harmless response in humans.

Due to the time spent in pre-clinical and clinical trials in the context of safety for humans, the ideal model at the present moment would be based on known and/or safe marketed VLPs that could be directed against these three diseases in a formulation system. Based on the already-known mechanisms of VLP technology and the deep understanding of the immune response to these parasites, it is still a utopian model of how this strategy can act in practice. 

Here, we propose VLPs that carry antigens of a single individual parasite, thereby becoming chimeric VLPs. According to some studies, intramuscular application has the highest efficiency [16], as it penetrates the epidermis to reach deeper layers, with a slow and gradual release of the formulation. Upon entering the organism, VLPs will be recognized as foreign bodies by APCs (antigen-presenting cells) that will mediate responses resulting from the formulation presented. In the ideal model, VLPs in association with antigens are processed and presented by MHC class II molecules and cause the stimulation of CD4^+^ T-helper lymphocytes, which will activate B cells and, in turn, stimulate the production of specific IgG antibodies.

When processed by APCs and presented by MHC class I to CD8^+^ T cells, a cytotoxic induction will be observed. Another expected response would be after the recognition of these antigens by CD4^+^ T cells: there will be an intensification in the production of cytokines, such as interferon-γ and TNF-α, which will induce a more aggressive response by macrophages with a high production of ROS and activate CD8^+^ T cells [71].

## 5. Final Considerations and Conclusions

As presented above, VLPs have contributed to the development of new vaccines to control numerous pathogens of medical importance. Therefore, we believe that this technology is promising as a vaccine adjuvant for the manipulation of the immune response during the induction of immunity, mainly in the context of complex diseases, such as infections caused by trypanosomatids. Finally, VLP-based vaccines, when properly designed and formulated, represent a promising platform for chemical or genetic conjugation of parasitic antigens and the development of multivalent vaccines against protozoa.

## Data Availability

Not applicable.
